# The complete chloroplast genome sequence of *Veratrum oxysepalum* and phylogenetic analysis

**DOI:** 10.1080/23802359.2021.1940330

**Published:** 2021-06-15

**Authors:** Zi-Jian Chen, Jia-Jia Liu, Ying-Min Zhang, Zi-Gang Qian, Guo-Dong Li

**Affiliations:** a Yunnan Key Laboratory for Dai and Yi Medicines, Yunnan University of Chinese Medicine, Kunming, Yunnan, People's Republic of China; b Faculty of Traditional Chinese Pharmacy, Yunnan University of Chinese Medicine, Kunming, People's Republic of China

**Keywords:** *Veratrum oxysepalum*, chloroplast genome, medicinal plant, phylogenetic analysis

## Abstract

*Veratrum oxysepalum* Turcz. is a medicinal plant belonging to Melanthiaceae occurring in Northeast China. However, there are still limited genomic resources available for genus *Veratrum*. The complete chloroplast (cp) genome of *V. oxysepalum* was determined and analyzed in this study. The complete cp genome was 153,705 bp. That contains a large single copy (LSC) region of 83,384 bp, a small single copy (SSC) region of 17,607 bp, which were separated by a pair of 26,358 bp inverted repeat regions (IRs). A total of 135 genes were annotated, including 83 protein-coding genes, 38 tRNAs, and eight rRNAs. Phylogenetic analysis using total chloroplast genome sequence of 21 species revealed that *V. oxysepalum* was closely relates to *V. patulum* of *Veratrum* with 100% bootstrap value.

There are approximately 17–45 species of perennial herbaceous plants of the genus *Veratrum L*. in the world. (Zomlefer et al. 2003). *Veratrum oxysepalum* Turcz., a perennial herb belonging to the genus *Veratrum.* It has widely distributed in hillside forest and wet meadow in Liaoning, Jilin and Heilongjiang provinces of China (Editorial Committee of Chinese Academy of Sciences 1980). *V. oxysepalum* was usually treated as traditional Chinese herbal medicines, its roots and rhizomes used to treat aphasia symptoms arising from apoplexy, wind type dysentery, jaundice, head-ache, scabies, chronic malaria and other disorders (Cong et al. 2020). In addition, genus *Veratrum* contains toxic compounds such as ester-alkaloids, there are numerous cases of mistaken identity that have resulted in *Ver*atrum poisoning (Christopher and McDougal 2014). For the safety of medication, it is necessary to accurately identify the species *Veratrum*. Here, we reported the complete chloroplast genome of *V. oxysepalum* to provide genomic resource for further conservation genetics and phylogenetic analysis.

The plant sample were collected from Jilin (43°24′N, 126°38′E), Jilin Province of China, and voucher specimens (220007009) were deposited in Herbarium of Yunnan University of Chinese Medicine. Firstly, total genomic DNA was isolated from liquid nitrogen frozen and ground leaf material using the plant DNA extraction kit (Bioteke Corporation, China) and sequencing was performed on the Illumina HiSeq 2500 platform (Illumina, San Diego, CA). Secondly, we assembled the clean data of 3 Gb based on NOVOPlasty (Dierckxsens et al. 2017). The assembled complete chloroplast genome was annotated with the online annotation tool DOGMA (Wyman et al., 2004), and corrected manually with the Geneious R11 11.1.5 (Biomatters Ltd., Auckland, New Zealand).

The circular chloroplast genome of *V. oxysepalum* was 153,705 bp (GenBank accession number: MW147219) in length, contained a large single-copy (LSC) region of 83,384 bp, a small single-copy (SSC) region of 17,607 bp, separated by a pair of inverted repeat (IR) regions of 26,358 bp each. A total of 135 genes were annotated, including 83 protein-coding genes, 38 tRNAs, and eight rRNAs. The overall GC content of the cp genome, LSC, SSC and IR regions are 37.7%, 35.7%, 31.4%, 42.9%, respectively.

To further investigate its phylogenetic position of *V. oxysepalum*, plastome of 21 representative species were downloaded from NCBI GenBank database, *Smilax china* and *Lilium henryi* were selected as the outgroup to construct the plastome phylogeny. All of genomes were fully aligned using MAFFT (Katoh and Standley 2013). The phylogeny was using with RAxML (Stamatakis 2014), bootstrap probability values were calculated from 1000 replicates for supporting the branches evaluated under the GTR model. The phylogenetic tree shows that *V. oxysepalum* and *V. patulum* from Melanthiaceae formed a monophyletic clade with 100% bootstrap value (Figure 1). In conclusion, this study identified unique characteristics of the *V. oxysepalum* cp genome providing valuable information for further investigations on species identification and the phylogenetic evolution between *V. oxysepalum* and related species.

**Figure 1.**
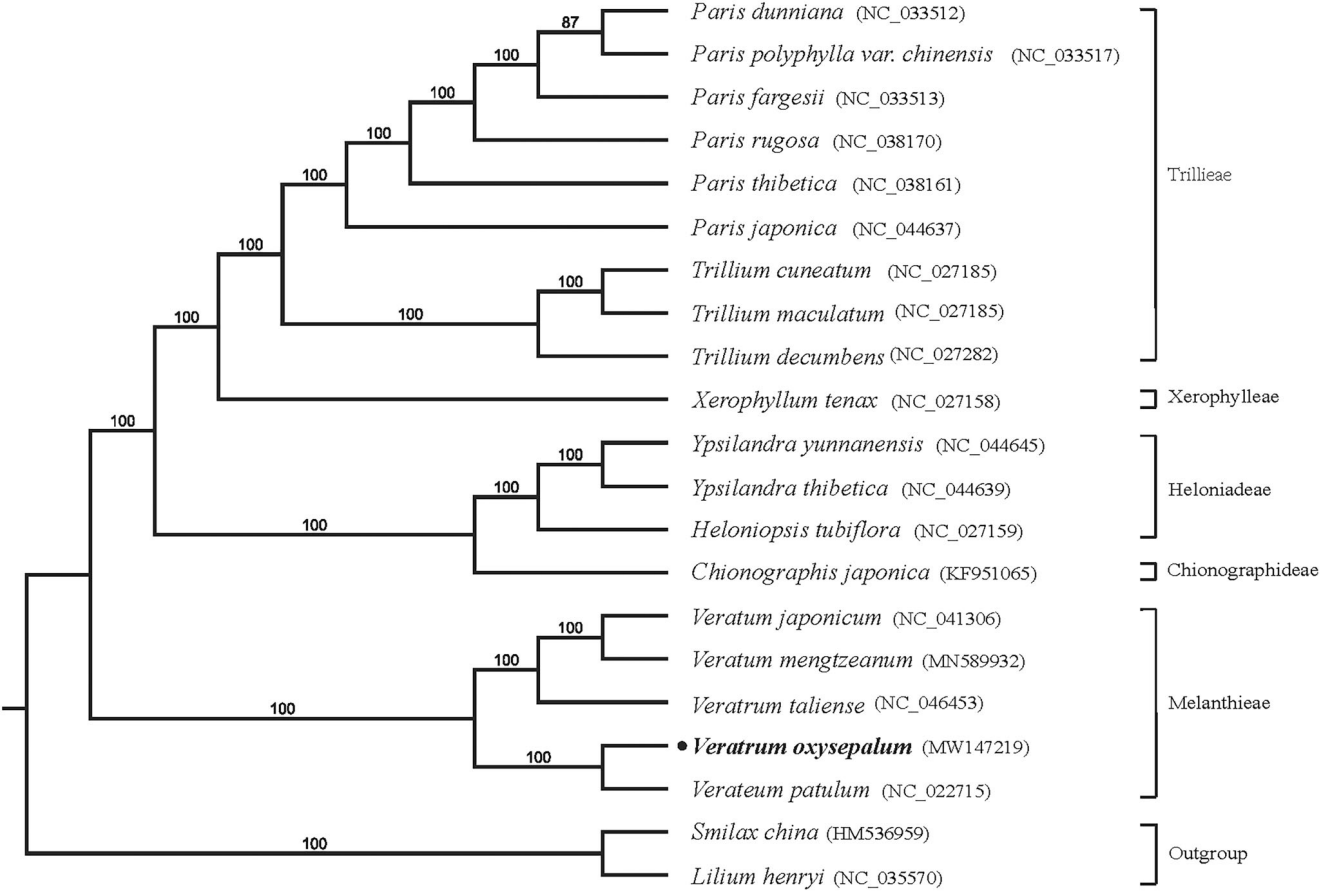
Maximum likelihood phylogenetic tree inferred from 21 chloroplast genomes. Bootstrap support values >50% are indicated next to the branches.

## Data Availability

The *Veratrum oxysepalum* data has been stored in nucleotide database of National Center of Biotechnology Information. GenBank accession number is MW147219. The SRA number is SRR14227219. All the information can be found on the website (https://www.ncbi.nlm.nih.gov/nuccore/).
